# Infant Skin Bacterial Communities Vary by Skin Site and Infant Age across Populations in Mexico and the United States

**DOI:** 10.1128/mSystems.00834-20

**Published:** 2020-11-03

**Authors:** Melissa B. Manus, Sahana Kuthyar, Ana Gabriela Perroni-Marañón, Alejandra Núñez-de la Mora, Katherine R. Amato

**Affiliations:** a Department of Anthropology, Northwestern University, Evanston, Illinois, USA; b Centro de Estudios de Opinión y Análisis, Universidad Veracruzana, Xalapa, Veracruz, México; c Instituto de Investigaciones Psicológicas, Universidad Veracruzana, Xalapa, Veracruz, México; UT Southwestern Medical Center at Dallas

**Keywords:** human microbiome, infancy, microbial ecology, skin microbiome

## Abstract

Daily practices put humans in close contact with the surrounding environment, and differences in these practices have an impact on human physiology, development, and health. There is mounting evidence that the microbiome represents an interface that mediates interactions between the human body and the environment. In particular, the skin microbiome serves as the primary interface with the external environment and aids in host immune function by contributing as the first line of defense against pathogens. Despite these important connections, we have only a basic understanding of how the skin microbiome is first established, or which environmental factors contribute to its development. To this end, this study compared the skin bacterial communities of infants (*n* = 47) living in four populations in Mexico and the United States that span the socioeconomic gradient, where we predicted that variation in physical and social environments would shape the infant skin microbiome. Results of 16S rRNA bacterial gene sequencing on 119 samples (armpit, hand, and forehead) showed that infant skin bacterial diversity and composition are shaped by population-level factors, including those related to socioeconomic status and household composition, and vary by skin site and infant age. Differences in infant-environment interactions, including with other people, appear to vary across the populations, likely influencing infant microbial exposures and, in turn, the composition of infant skin bacterial communities. These findings suggest that variation in microbial exposures stemming from the local environment in infancy can impact the establishment of the skin microbiome across body sites, with implications for developmental and health outcomes.

**IMPORTANCE** This study contributes to the sparse literature on the infant skin microbiome in general, and the virtually nonexistent literature on the infant skin microbiome in a field setting. While microbiome research often addresses patterns at a national scale, this study addresses the influence of population-level factors, such as maternal socioeconomic status and contact with caregivers, on infant skin bacterial communities. This approach strengthens our understanding of how local variables influence the infant skin microbiome, and paves the way for additional studies to combine biological sample collection with questionnaires to adequately capture how specific behaviors dictate infant microbial exposures. Work in this realm has implications for infant care and health, as well as for investigating how the microbial communities of different body sites develop over time, with applications to specific health outcomes associated with the skin microbiome (e.g., immune system development or atopic dermatitis).

## Abstract (precis)

This study contributes to the sparse literature on the infant skin microbiome in general, and the virtually nonexistent literature on the infant skin microbiome in a field setting. While microbiome research often addresses patterns at a national scale, this study addresses the influence of population-level factors, such as maternal socioeconomic status and contact with caregivers, on infant skin bacterial communities. This approach strengthens our understanding of how local variables influence the infant skin microbiome, and paves the way for additional studies to combine biological sample collection with questionnaires to adequately capture how specific behaviors dictate infant microbial exposures. Work in this realm has implications for infant care and health, as well as for investigating how the microbial communities of different body sites develop over time, with applications to specific health outcomes associated with the skin microbiome (e.g., immune system development or atopic dermatitis).

## INTRODUCTION

Human-environment interactions have a marked impact on host physiology, development, and health. There is mounting evidence that the human microbiome—the vast collection of microorganisms (and their genes) that live in, on, and around us— represents one mechanism through which interactions with the environment impact the human body. For example, the microbiome is shaped by exposure to external factors, including antibiotics (1), diet (2), and social interactions (3–5). In turn, microbial communities influence host nutrition (6, 7), play a vital role in proper immune system development in early life (8, 9), and can influence mood and behavior (10).

However, the majority of microbiome research is conducted on the gut and overlooks the organ that serves as the primary interface with the external environment—the skin. Microbial communities on the skin aid host immune function by contributing as the first line of defense against pathogens. For example, resident skin microbes, such as Staphylococcus epidermidis, exhibit anti-inflammatory properties and help inhibit the colonization of potential pathogens (11). Studies in mice highlight the role of commensal skin microbes in cytokine production and normal immune functioning (12), and these skin microbe-induced immune functions may have systemic effects on the body via the skin-gut axis (13–15).

Despite the appreciation that the skin microbiome sits at the interface of human-environment interactions and is linked to host physiology and health, we still have a nascent understanding of its initial development. As the in-utero environment is largely sterile, infants receive their first significant exposure to microbes at birth (16). By adulthood, humans have distinct microbial communities across different body sites (17). However, it is unclear how the relatively homogeneous newborn microbiome diverges into body site-specific microbial communities in the first 1,000 days of life (8, 18). Here, an ecological perspective is useful for understanding how behaviors influence how we acquire microbes from the environment. For example, microbes from the birth environment, including from the mother’s body, are available for dispersal to the newborn during delivery and initial physical contact between mothers and newborns (16, 19–21). Further, the skin microbiome may aid in transferring microbes from the environment to different sites across the body, including the gut. This may be especially relevant for infants, where common behaviors such as putting objects and body parts into their mouths may promote the skin’s role as a “microbial vector,” transmitting microbes from the environment to the skin, and into the gut. Thus, the skin may be an important, but currently underexplored, component of the developmental trajectory of the overall infant microbiome.

While studies in nonhuman primates and human infants suggest that the environment, including the social environment, has marked impacts on the gut microbiome (22–27), few studies have quantified the influential role of early life social environments and their effects on infant skin microbial communities. Because the adult skin microbiome is affected by contact with other people (28, 29), nonhuman animals (30), surfaces (31, 32), and hygiene product use (33, 34), it is likely that variation in these factors during early life shapes the development of the infant skin microbiome (35). However, early life environments differ across human populations, suggesting that an infant’s exposure to environmentally sourced microbes, including those from the social environment, may be highly variable across settings. For example, differences in childcare practices across sociocultural contexts could contribute to differential microbial exposures, including those related to attending daycare (36), hygiene practices (34), and physical contact with siblings or other caretakers (37–42).

To address the gaps in the literature surrounding the early life skin microbiome, we conducted a cross-sectional study that explored the relationship between early life environments and the skin bacterial communities of infants. Here, we use the term “bacterial” to refer directly to the results of this study, and use the terms “microbiome,” “microbe,” and “microbial” when discussing trends in the literature. As geography often serves as a proxy for dietary and lifestyle variation (43), we recruited participants in Evanston, IL, USA and in three different populations in Veracruz, Mexico (Xalapa, Coatepec, and Ocotepec) to capture lifestyle variation both within and between populations. Details of each population can be found in the Materials and Methods section. Moving forward, we will refer to the populations as follows: Evanston as “urban USA”; Xalapa as “urban MEX”; Coatepec as “peri-urban MEX”; and Ocotepec as “rural MEX” (Table 1). By coupling skin bacterial samples with questionnaire data, we were able to compare the effects of external variables, such as geography (43) and household composition (38), as well as intrinsic host factors, like age (8), on skin bacterial communities in early life. To our knowledge, this is one of the few studies to collect skin bacterial samples in a field setting (defined here as research outside a clinical or home environment, without participant self-collection), and the first study to compare skin bacterial samples from infants living in different geographic and socioeconomic contexts. Because geography is associated with lifestyle variation, we hypothesized (i) there would be differences in infant skin bacterial communities across the populations. More specifically, we expected that this variation would covary with variables related to socioeconomic status and household environments (38, 40). Since different body sites harbor distinct microbial communities (17), we also hypothesized that (ii) there would be differences in the diversity of infant skin bacteria across the sampled skin sites. Additionally, physiological properties of the skin change throughout infancy (e.g., hydration levels [44] and pH [45]), which likely affects which microbes persist at particular body sites (8). Therefore, we also expected (iii) infant age to be positively correlated with the diversity of skin bacterial communities, such that older infants will harbor more bacterial diversity and heterogeneity across skin sites.

**TABLE 1:** Population demographics

Population	Profile	Socioeconomic status	No. infants sampled
Evanston	Urban USA	High	25
Xalapa	Urban MEX	Middle	7
Coatepec	Peri-urban MEX	Low	7
Ocotepec	Rural MEX	Low	8

## RESULTS

Analyses were conducted on a total of 119 skin bacterial samples from 47 infants aged 0.5 to 33 months, with an average age of 11.8 months across the four populations (Table 2). All participants were sampled outside their homes, either in an office space in the United States or in an outdoor common area in Mexico. Despite the differences in sampling location, swabs only contacted the participants’ skin, and control samples of the ambient air returned no detectable bacterial signature. This makes an impact of collection environment on our results unlikely. Household size ranged from 3 to 14 people (including the infant), and the number of alloparents (i.e., caregivers other than the mother) ranged from 0 to 9, where the rural MEX population had the highest household size and number of alloparents (Table 2). Responses to questionnaires indicated that lifestyle factors likely to impact the infant skin microbiome differed across the populations, including hygiene practices and delivery mode. For example, infants were bathed less frequently in the rural MEX population compared to the other populations. The majority of infants in the rural MEX population were delivered vaginally (7 out of 8), while most in the urban MEX population were delivered via Cesarean section (6 out of 7). Delivery mode was more variable in the other two populations, with about half of the infants in the peri-urban MEX population (3 out of 7) and one-third in the urban USA population (7 out of 25) delivered by Cesarean section.

**TABLE 2:** Samples used in statistical analyses, including the number of skin samples per population, the age range of infants, the range of household size (including the infant), and the range of alloparents reported per infant

Population	No. skin samples	Age (mo)	Mean age (mo)	Household size	Alloparents
All populations combined	119	0.5–33	11.8	3–14	0–9
Urban USA	65	5–13	10.2	3–5	0–4
Urban MEX	18	3–33	15.7	3–4	1–3
Peri-urban MEX	14	0.5–18	8.8	3–7	1–3
Rural MEX	22	2.5–32	12.9	3–14	0–9

### Predictors of bacterial community composition.

To identify characteristics that predicted skin bacterial community composition, we conducted variance analyses on UniFrac distances. In the overall models of both unweighted and weighted UniFrac distances, we found that population, body site, infant age, household size, number of alloparents, and delivery mode were all significantly associated with variation in skin bacterial communities across all samples, though number of siblings was not (Table 3). This held true for the subsetted models that isolated potentially correlated predictors (e.g., household size and number of alloparents; see the Materials and Methods), with the exception of delivery mode in certain models (Table S1 in the supplemental material).

**TABLE 3:** Results of PERMANOVA on unweighted and weighted UniFrac distances (all samples combined)

Parameter	df	pseudo-F	*R*^2^	*P* value^*a*^
Unweighted UniFrac distance				
Body site	2	3.703	0.052	**<0.001**
C-section	1	1.987	0.014	**<0.01**
Infant age	1	4.034	0.028	**<0.001**
Siblings	1	1.239	0.009	0.185
Household size	6	1.534	0.064	**<0.001**
Alloparents	6	1.598	0.066	**<0.001**
Population	3	1.888	0.039	**<0.001**
Weighted UniFrac distance				
Body site	2	20.337	0.219	**<0.001**
C-section	1	2.334	0.013	**<0.05**
Infant age	1	3.105	0.017	**<0.05**
Siblings	1	1.670	0.009	0.113
Household size	6	1.972	0.064	**<0.01**
Alloparents	6	2.003	0.065	**<0.01**
Population	3	3.103	0.050	**<0.001**

a *P* values in boldface indicate significance.

10.1128/mSystems.00834-20.1 TABLE S1 PERMANOVA on metrics of beta diversity (all samples combined). Download Table S1, DOCX file, 0.1 MB. Copyright © 2020 Manus et al. 2020 Manus et al. This content is distributed under the terms of the Creative Commons Attribution 4.0 International license.

**FIG 1:**
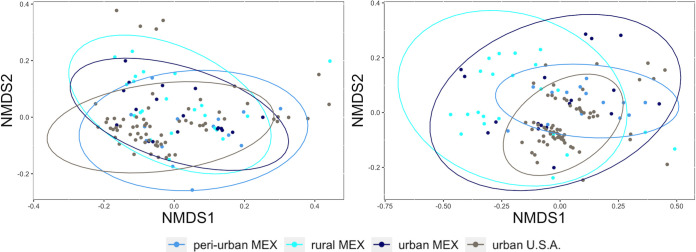
NMDS plots displaying samples by population: weighted UniFrac distances (left) and unweighted UniFrac distances (right).

**FIG 2:**
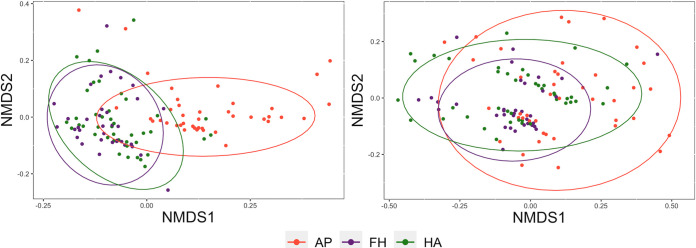
NMDS plots displaying samples by body site: weighted UniFrac distances (left) and unweighted UniFrac distances (right) (AP = armpit, FH = forehead, HA = hand).

Nonmetric multidimensional scaling (NMDS) plots show that the samples cluster by body site more clearly than by population (Fig. 1 and Fig. 2). Pairwise PERMANOVA of unweighted UniFrac distances yielded significant differences between all comparisons of populations, skin sites (armpits-foreheads, armpits-hands, foreheads-hands), and infant age groups (Table 4). Tests of weighted UniFrac values showed significant differences between rural MEX and urban USA, two of the three body site comparisons (hands-armpits and armpits-foreheads), and the age groups (Table 4). The effect sizes of the significant body site comparisons using weighted UniFrac distances were considerably larger than the same comparisons using unweighted UniFrac distances.

**TABLE 4:** Results of pairwise PERMANOVA tests (all samples combined)

Comparison	df	pseudo-F	*R*^2^	*P* value^*a*^
Between populations				
Unweighted UniFrac				
Peri-urban MEX versus urban USA	1	3.173	0.040	**<0.01**
Rural MEX versus peri-urban MEX	1	3.926	0.104	**<0.01**
Peri-urban MEX versus urban MEX	1	1.672	0.053	**<0.05**
Rural MEX versus urban USA	1	7.761	0.084	**<0.01**
Urban MEX versus urban USA	1	3.408	0.040	**<0.01**
Rural MEX versus urban MEX	1	2.173	0.054	**<0.05**
Weighted UniFrac				
Peri-urban MEX versus urban USA	1	2.480	0.031	0.142
Rural MEX versus peri-urban MEX	1	2.388	0.066	0.142
Peri-urban MEX versus urban MEX	1	0.942	0.030	0.519
Rural MEX versus urban USA	1	4.532	0.051	**<0.01**
Urban MEX versus urban USA	1	2.769	0.033	0.113
Rural MEX versus urban MEX	1	1.242	0.066	0.512
Between body sites^*b*^				
Unweighted UniFrac				
HA-AP	1	3.526	0.041	**<0.001**
HA-FH	1	2.095	0.027	**<0.01**
AP-FH	1	4.774	0.060	**<0.001**
Weighted UniFrac				
HA-AP	1	23.598	0.223	**<0.001**
HA-FH	1	1.439	0.019	0.160
AP-FH	1	28.762	0.277	**<0.001**
Between infant age groups^*c*^				
Unweighted UniFrac				
Older-younger	1	3.037	0.025	**<0.001**
Weighted UniFrac				
Older-younger	1	3.966	0.033	**<0.01**

a *P* values in boldface indicate significance.

b HA = hands, AP = armpit, FH = forehead.

c Older group = 7 to 33 months, younger group = 0 to 6 months.

### Differences in bacterial taxonomic richness by body site.

**FIG 3:**
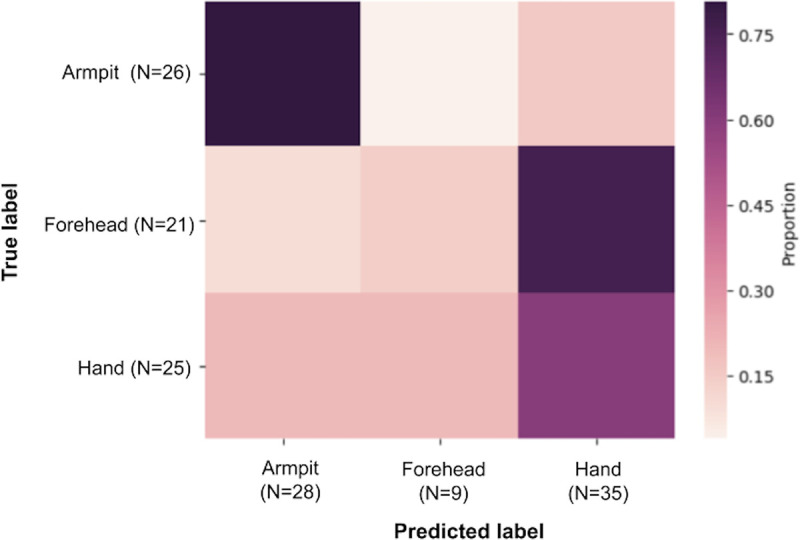
Heatmap of algorithm-based classifier results by body site. Accuracy of classification was 80% for armpit samples, 60% for hand samples, and 14% for forehead samples.

Overall, we found that samples from infants were dominated by four bacterial phyla (*Firmicutes*, *Actinobacteria*, *Proteobacteria*, and *Bacteroidetes*; Fig. S1), and that the algorithm-based classifier performed with an overall accuracy of 0.54 when classifying samples by body site (Fig. 3). To further explore differences in bacterial diversity by skin site, we focused subsequent analyses on models of alpha diversity at the amplicon sequence variant (ASV) level (Faith’s phylogenetic distance [PD] is reported here; Shannon diversity is reported in Table S2). We first ran ANOVA analysis on linear mixed effect models with all samples combined, which showed that samples from infants in the rural MEX population displayed significantly higher skin alpha diversity than those in the peri-urban MEX population (estimate = 21.061, *P* < 0.001), the urban USA population (estimate = 18.223, *P* < 0.001), and the urban MEX population (estimate = 11.859, *P* < 0.05). Models of Shannon index yielded similar results (Table S2).

10.1128/mSystems.00834-20.6 FIG S1 Percentage relative abundance of bacterial phyla across body sites; “Other” phyla is a summed value of all bacterial phyla with a percent relative abundance ≤ 2%. Download FIG S1, TIF file, 1.5 MB. Copyright © 2020 Manus et al. 2020 Manus et al. This content is distributed under the terms of the Creative Commons Attribution 4.0 International license.

10.1128/mSystems.00834-20.2 TABLE S2 ANOVA on linear mixed effects models of alpha diversity (Shannon index). Download Table S2, DOCX file, 0.1 MB. Copyright © 2020 Manus et al. 2020 Manus et al. This content is distributed under the terms of the Creative Commons Attribution 4.0 International license.

**FIG 4:**
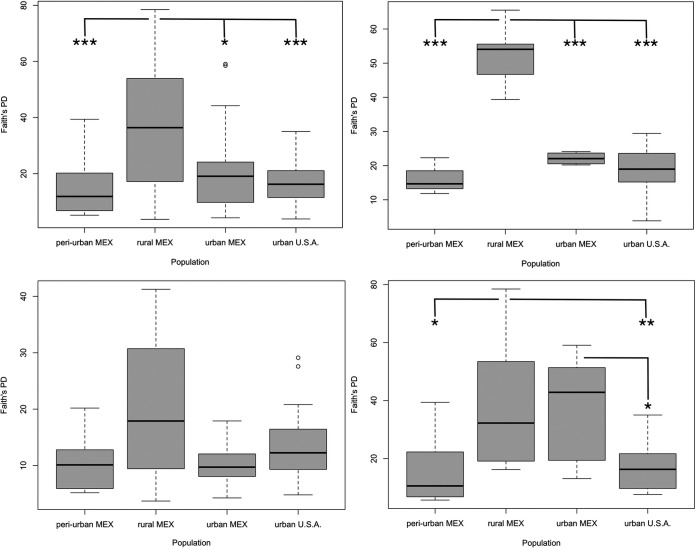
Differences in bacterial diversity across the populations vary by body site (ANOVA on Faith’s PD). Clockwise from top left: all skin samples combined; forehead samples; armpit samples; hand samples (* = *P* < 0.05, ** = *P* < 0.01, *** = *P* < 0.001).

Due to the large effect of body site in the models of beta diversity, as well as the variation in the accuracy of the classifier by each of the three body sites (Fig. 3), we ran the same models of bacterial diversity on samples separated by body site. The trend of elevated diversity in the rural MEX population persisted when the model was restricted to only forehead samples (rural MEX/peri-urban MEX estimate = 39.274; rural MEX/urban USA estimate = 33.282; rural MEX/urban MEX estimate = 30.449; all comparisons *P* < 0.001). Models of alpha diversity (Faith’s PD) in hand samples yielded differences between the rural and peri-urban MEX populations (estimate = 21.094, *P* < 0.05), the rural MEX and urban USA populations (estimate = 20.742, *P* < 0.01), and the urban MEX and urban USA populations (estimate = 19.341, *P* < 0.05), but not between the rural MEX and urban MEX populations (estimate = 1.401, *P* = 0.998). Results of the same models using Shannon index are reported in Table S2. All comparisons are illustrated in Fig. 4. Results from linear discriminant analysis effect size (LEfSe analysis) indicate that hand samples from infants in the urban MEX population harbored almost as many discriminative bacterial ASVs as the infants from rural MEX (21 versus 31) (Table S3), including soil-derived microbes (e.g., *Massilia* [46], Bacillus fumarioli [47], and Pseudomonas nitroreducens [48]). Similarly, samples from infants in the rural MEX population contained microbes associated with domesticated animals (e.g., Streptococcus alactolyticus [49, 50]) as well as plants (e.g., Methylobacterium mesophilicum [51]). There were no differences in armpit samples across the populations (using either index). Accordingly, the algorithm-based classifier accurately classified armpits the most frequently (∼80%), followed by hands (∼60%), and then foreheads (∼14%) (Fig. 3).

10.1128/mSystems.00834-20.3 TABLE S3 Number of discriminative bacteria at the ASV taxonomic level in each population. Download Table S3, DOCX file, 0.04 MB. Copyright © 2020 Manus et al. 2020 Manus et al. This content is distributed under the terms of the Creative Commons Attribution 4.0 International license.

Using linear mixed effects models to test for the relative abundance of particular bacterial ASVs in the data set (*n* = 179 ASVs), we found that the relative abundance of 11 different ASVs varied significantly by population (corrected *P* value <0.01) (Table 5). The relative abundance of 45 ASVs varied by body site, while number of alloparents and household size were significantly associated with the relative abundance of 8 and 12 ASVs, respectively. The specific ASVs are listed in Table S4.

**TABLE 5:** Results of ANOVA on linear mixed effects models testing the influence of independent variables on the relative abundance of bacterial ASVs (all samples combined)

Independent variable (*n* = 119 samples)	No. of differentially abundant bacterial ASVs (out of 179 total ASVs)^*a*^
Population	11 (6%)
Body site	45 (25%)
No. of alloparents	8 (5%)
Household size	12 (7%)

a Models were run on ASVs with relative abundance counts of ≥500 across the whole data set. Percentages in the parentheses indicate the percentage of significantly variable ASVs out of the 179 total ASVs in the data set.

10.1128/mSystems.00834-20.4 TABLE S4 Bacterial ASVs with relative abundances that are significantly associated with population, body site, number of alloparents, and household size; mean relative abundance of each ASV (per sample) is listed for each factor of the categorical variables (population and body site), along with the test statistic of the linear mixed effects models. Download Table S4, DOCX file, 0.1 MB. Copyright © 2020 Manus et al. 2020 Manus et al. This content is distributed under the terms of the Creative Commons Attribution 4.0 International license.

### Differences in bacterial taxonomic richness emerge in the older age group.

Since age had a large effect in models of beta diversity, we also explored the effect of age on alpha diversity. In the rural MEX population, there were no differences in alpha diversity across skin sites in the younger age group, while foreheads harbored more bacterial taxa compared to armpits in the older infants (estimate = 27.929, *P* < 0.01). In the peri-urban MEX population, there were no differences between skin sites within either age group. A skewed age distribution toward older infants limited our ability to model the younger age group on its own in both urban populations. However, the hands of older infants in the urban MEX population were more diverse than their armpits (estimate = 31.831, *P* < 0.001) and foreheads (estimate = 21.461, *P* < 0.01). Finally, foreheads (estimate = 5.597, *P* < 0.001) of older infants harbored increased bacterial diversity compared to armpits in the urban USA population. Table 6 illustrates the results of linear mixed methods models on samples from infants in the older age group (Shannon index data are in Table S5).

**TABLE 6:** Results of ANOVA on linear mixed effects models comparing Faith’s PD across body sites in the older age group (7 to 33 months)^*a*^

Population	FH-AP (test estimate, *P* value)	HA-AP (test estimate, *P* value)	HA-FH (test estimate, *P* value)
Urban USA	5.597, ***P* < 0.001**	3.476, *P* = 0.060	−2.121, *P* = 0.325
Urban MEX	10.370, *P* = 0.230	31.831, ***P* < 0.001**	21.461, ***P* < 0.01**
Peri-urban MEX	5.813, *P* = 0.771	−0.068, *P* = 1.000	−5.881, *P* = 0.766
Rural MEX	27.929, ***P* < 0.01**	13.222, *P* = 0.186	−14.707, *P* = 0.153

a AP = armpit, FH = forehead, HA = hand. *P* values in boldface indicate significance.

10.1128/mSystems.00834-20.5 TABLE S5 Results of ANOVA (test estimate and *P* value) on linear mixed effects models comparing Shannon index across body sites in the older age group (7 to 33 months). Download Table S5, DOCX file, 0.04 MB. Copyright © 2020 Manus et al. 2020 Manus et al. This content is distributed under the terms of the Creative Commons Attribution 4.0 International license.

## DISCUSSION

This study examined the relationship between infants’ early life environments and the diversity and composition of their skin bacterial communities. In support of our hypotheses, our results showed that differences in infant skin bacterial diversity based on geography are population specific, and vary by skin site and infant age. Importantly, our study probes deeper than the national level by including multiple Mexican populations, and thus highlights differences in the infant skin microbiome that emerge at the population level. In contrast to previous studies (including on the microbiomes of body sites other than the skin) that attribute differences in microbial communities to lifestyle variation based on broad national, ethnic, or “racial” categories (43, 52–55), our study quantified population-level factors that may influence early life microbial environments. Our results highlight the need for a more nuanced approach in the burgeoning skin microbiome literature, including the consideration of particular behaviors that put individuals in contact with the environment (and its microbes). For example, significant predictors in our models, including household size and number of alloparents, suggest routes by which infants differentially come into contact with microbes from diverse physical and social environments, even within the same country. Moving forward, studies across socioeconomic settings within a given geographical area are well suited to capture variation in lifestyle factors that may impact the microbial environment of infancy.

### Skin bacterial diversity differs across populations.

We found differences in skin bacterial composition and diversity across the four populations. Samples from infants in the rural MEX population displayed elevated bacterial diversity, which could be due to increased exposure to microbes from the natural environment, including from soil, plants, and other animals (56, 57). Samples from these infants harbored environmentally derived taxa, including the freshwater bacterium Inhella inkyongensis (58), the soil bacteria Parasegitibacter luojiensis (59) and *Limnobacter* spp. (60), and *Acidovorax* spp., a bacterial group known to cause disease in crops (61). Because our environmental controls did not contain detectable levels of bacteria, these patterns are unlikely to be a result of contamination. It may be that infants in this agricultural population are in direct contact with these microbes (e.g., from soil and natural water sources), and/or that caregivers transfer microbes from the environment to infants via physical contact (38). In general, more work is needed to delineate between microbes that are true residents of the skin versus those that reflect periodic exposures to microbes from the natural environment. In settings like the rural MEX population, frequent exposure to the natural environment may result in “environmental” microbes acting as more persistent community members on infant skin compared to settings where environmental exposures are less frequent and/or hygiene practices are more stringent. Moreover, processes like horizontal gene transfer may allow transient environmental microbes (i.e., those that do not successfully colonize infant skin) to alter the genetic and functional properties of the existing skin bacterial community without changing the taxonomic composition (62). Since the skin microbiome plays a role in regulating the immune system (12), functional changes to skin microbial communities that arise via horizontal gene transfer may affect early life immune system development and, in turn, lifelong health. Further, if infant behaviors promote the spread of skin bacteria into the gut (e.g., putting fingers into the mouth), then the external microbial environment may indirectly shape the infant gut bacterial community via the skin.

Although we did not directly measure infant bathing or ask about the time since last bath on the questionnaire, we did capture basic information about bathing practices across the populations that may affect bacterial skin communities. For example, while infants in the rural MEX population are bathed less frequently than infants in the other populations (a few days per week compared to every day), bathing in this setting may actually expose infants to more environmentally sourced microbes through contact with natural water sources (34). Further, we found some support that delivery mode affects skin bacterial community composition (16), though this varied across models. Future studies would benefit from repeatedly sampling infants in the first weeks to months of life, as this would help elucidate the extent to which microbial exposures at delivery are “counteracted” by exposures later in life (8).

Mothers in the rural MEX population reported the largest household sizes and the greatest number of alloparents, suggesting that infants in this population are exposed to microbially rich environments through contact with alloparents and/or other people in the household, in addition to exposure to the physical environment. This trend is in line with existing evidence for a relationship between household composition and infant gut bacterial taxonomic composition, including bacterial taxa like *Lactobacillus*, *Klebsiella*, *Clostridium*, and *Enterobacter* (38). Though this study did not directly test the transfer of microbes from caregivers to infants, both household size and number of alloparents were significant predictors of infant skin bacterial composition and were significantly associated with the relative abundance of particular bacterial ASVs. While the linear mixed effects models on the relative abundance of these ASVs do not indicate the direction of the relationship (i.e., if infants in larger households harbor more of a particular ASV), these trends are an important first step for additional studies that make predictions about the relative abundance of certain microbes based on host attributes, or to target specific bacterial taxa for strain-level analyses. Our study contributes to the growing literature on social dynamics and the microbiome in both nonhuman primates (4, 5, 23, 26) and humans (37–39, 63, 64), and highlights how multiple socioeconomic factors, like mother’s education and number of alloparents, may work in tandem to influence infants’ physical, social, and microbial environments.

Interestingly, the number of siblings in the household did not predict infant skin bacterial composition, while other studies have found that having older siblings is correlated with decreased bacterial diversity (40–42). However, our questionnaire did not capture sibling age, making it difficult to infer how the infants in our study interact with their siblings. For example, it may be that siblings of infants in our study were not yet at the age to be contributing substantial allocare, which would limit the amount of intersibling bacterial sharing. Further, our questionnaire did not quantify time spent playing between siblings, which may serve as another route for bacterial sharing between siblings. Additionally, older infants in our study may come into contact with other children who are not siblings, though our questionnaire did not address these interactions. Future work on the social transmission of microbes to infants would benefit from documenting the frequency of contact with alloparents and other members of the social environment, including siblings.

### Comparisons of skin bacterial diversity vary by skin site.

Differences in skin bacterial diversity across the populations varied by skin site, which is in line with the robust literature on variation in microbial community diversity and composition across sites on the skin (8, 17, 29, 52, 65, 66). For example, the elevated bacterial diversity in the rural MEX population compared to the other populations was driven by forehead samples. This may be a result of increased contact with microbes from caregivers (i.e., kissing and touching the forehead), as the largest number of alloparents per infant were reported by mothers in this population. Moreover, it may be that as a sebaceous skin site (67), the infant forehead is able to harbor skin bacteria that originate from the faces of caregivers. In this scenario, behaviors that put caregivers into face-to-face contact with infants, such as kissing and nestling heads, may promote bacterial sharing and increase the taxonomic diversity of the infant forehead compared to body sites that are less frequently contacted. If the combination of human behavior and ecological properties of the skin results in increased bacterial diversity on the infant forehead, this may drive greater interindividual bacterial variation in forehead samples. In turn, this variability may explain why these samples were poorly identified by the algorithm-based classifier.

In contrast, armpit samples did not display different levels of bacterial diversity across the populations. As armpits are relatively protected from the outside environment—both by their anatomical position and the protection provided by clothing—compared to other body sites, they may be less influenced by contact with environmentally sourced microbes. Every infant in our study was clothed immediately prior to sample collection, and many wore coats (e.g., infants from the rural MEX and urban USA populations who were sampled in colder weather) or were wrapped in blankets (e.g., younger infants). Based on these observations, it is likely that the armpits of many infants in our study are frequently protected from the outside environment, though additional sampling during warmer weather would help to elucidate the role of clothing as a barrier to microbial exposure. This reduction in environmental exposure may promote decreased interindividual variation in armpit samples, enabling the algorithm-based classifier to accurately identify armpit samples. Further, the armpit harbors less diverse microbial communities compared to other body sites (57, 65), in part due to the secretions of densely packed apocrine sweat glands that support the growth of certain bacteria (68, 69). In our study, it may be that similar properties dictate the reduced variation in armpit samples collected across the populations, though further research is needed to characterize if processes that affect bacteria, like sweat production, are as active in the infant armpit compared to the adult armpit. Additionally, it is likely that other behaviors shape infant armpit bacterial communities, such as being bathed or picked up by caregivers, which could increase opportunities for bacterial sharing between caregiver hands and infant armpits.

It is noteworthy that the effect sizes of the significant pairwise body site comparisons (hand-armpit and forehead-armpit) using weighted UniFrac distances were considerably larger than the same comparisons using unweighted UniFrac distances. Unlike unweighted UniFrac, the weighted metric does not bias toward “rare” or “minor” bacterial community members (70). Thus, these results may reflect distinctions in community composition driven by the more “major” microbes found in armpits versus the other two body sites. While skin properties that impact microbes, such as hydration levels (44) and pH (45), likely vary between infant and adult armpits (e.g., due to the presence of sweat glands and body hair in adults), our finding of decreased bacterial (alpha) diversity in armpit samples is in line with results of previous studies on adults both within and outside the United States (57, 65, 68). Overall, these findings support the idea that armpit samples are distinct and more homogeneous compared to the other body sites, which may explain why the results of the algorithm-based classifier were the most accurate for the armpit samples.

Variable patterns of environmental contact likely also explain why hand samples taken from the rural and urban MEX populations displayed higher bacterial diversity than those from the peri-urban MEX and urban USA populations. Infants in the urban MEX population have regular access to an outdoor play area, and were observed to be playing in grass and soil prior to sample collection. Frequent contact with soil and plant material may explain the similarity in bacterial diversity of hand samples in the rural and urban MEX populations, as well as the fact that hand samples from these populations harbored soil-derived microbes (albeit in relatively low abundances), including *Massilia* (46), Bacillus fumarioli (47), and Pseudomonas nitroreducens (48), as well as microbes associated with domesticated animals (e.g., Streptococcus alactolyticus [49, 50]) and plants (e.g., Methylobacterium mesophilicum [51]). Taken together, these results suggest that skin bacterial communities can reflect certain behaviors that put infants in contact with environmental microbes though, as previously mentioned, further research is needed to determine which environmentally derived taxa are transient members of the human skin versus those that become stable residents.

### Skin bacterial diversity is driven by older infants.

In line with previous work on the skin microbiome, samples from infants in both age groups in this study were dominated by *Firmicutes*, *Actinobacteria*, *Proteobacteria*, and *Bacteroidetes* (29, 52, 57, 65, 66, 71, 72). It is noteworthy that the differences in bacterial diversity across the skin sites are driven by individuals in the older age group (7 to 33 months) compared to the younger age group (0 to 6 months). This is in line with suggestions that newborns have relatively homogeneous microbiomes (16), with body site-specific differences emerging later in life (8). Because the current study was conducted on multiple populations, our results expand upon this finding by comparing the influence of age across different geographic and socioeconomic settings.

When restricting analyses to the group of older infants, the results of comparisons of bacterial diversity between skin sites varied across the populations, suggesting that patterns of skin site-specific bacterial communities unfold unequally in different environmental settings. These differences may be due to a combination of endogenous host properties and exogenous environmental factors. For example, changes in skin physiology, including hydration levels (44) and pH (45), may alter local environmental conditions that dictate which microbes are able to persist at particular body sites (17, 71). At the same time, behavioral and development changes in infancy (e.g., learning to crawl or playing with siblings) likely alter patterns of contact between the skin and environmentally sourced microbes, contributing to different environmental microbial exposures at different ages. Future work is needed to identify both intrinsic and extrinsic factors that affect skin microbial communities as infants grow older, and how this varies by body site.

To our knowledge, this is one of the few studies to use next-generation sequencing approaches to investigate the bacterial communities of the skin of healthy infants (8, 16, 73), and the first to collect skin samples in a field setting outside the United States. Our combination of biological sample collection and qualitative survey data across multiple populations, infant body sites, and infant ages allows for a more robust exploration of the variables that influence early life skin microbial communities. This study would benefit from more even sampling within each population, particularly in relation to infant age, as a skew toward older individuals in two of the populations limited certain analyses and restricted the distribution of the infant age groups. One possible result of the wide age range of the older group (7 to 33 months) is that behaviors of the eldest infants masked the behaviors, and associated microbial exposures, of younger infants that were placed in the same age group. For example, older toddlers who are learning to walk and feed themselves may be exposed to different microbes than are less mobile infants who are still breastfeeding. Similarly, differences in the frequency of washing clothing, both between age groups and populations, may contribute to variation in microbial exposures of infant skin.

Additionally, we only utilized one population in the United States in this study, which certainly did not capture the range of environmental and behavioral variation within the country. Further, our questionnaire did not quantify the frequency of specific behaviors that put infants into contact with the environment, including other people. Future studies would benefit from a more formal evaluation of how certain behaviors, like carrying, cosleeping, and breastfeeding, expose the infant skin to microbes. Finally, while the biomass of microbes in the ambient air at our sampling locations was likely low (74, 75), researchers should consider ways to better quantify environmental controls in future work (e.g., the use of an air pump to collect ambient air control samples, or collecting soil samples for comparison to skin samples), especially in field contexts where samples cannot be collected indoors in a sterilized setting.

Moving forward, there is a need for more infant skin microbiome samples collected across diverse geographic, socioeconomic, and cultural settings, particularly where differences in behaviors, such as those related to childrearing and hygiene, may lead to differential microbial exposures early in life. Continuing to incorporate cultural and socioeconomic differences that may affect the infant skin microbiome will allow for a more robust understanding of the range of “normal” microbial exposure and acquisition. Given calls for exploring the influence of the social environment on the microbiome (22, 76), our results suggest that future work in this area would benefit from longitudinal sampling as well as direct participant observation in order to capture how temporal, geographic, and behavioral variation impacts the development of the infant skin microbiome. Finally, because our results differed depending on if analyses were conducted with pooled samples or by each body site independently, we recommend the continued sampling and analyses of different body sites. This will lead to improved characterization of skin sites across the body, contributing to our understanding of the skin microbiome as a collection of diverse ecological niches that harbor different microbial communities (17).

More broadly, understanding how the early life environment shapes the infant skin microbiome can set the stage for future work that connects early life microbial communities to developmental and health outcomes. Because the skin microbiome is linked to immune system functioning (12, 35, 77), early life microbial acquisition may help prepare infants for both short- and long-term health challenges. Specifically, variation in early life environments may lead to differences in infant skin microbial acquisition within an early “critical window,” triggering unequal trajectories of immune system development and associated health outcomes (78). Results from the current study suggest that variation in microbial exposures during this period may stem from differences in lifestyle and behavior, highlighting the need for additional studies across diverse environmental settings. Such studies will help quantify the “microbial environment of infancy,” as well as identify the potential for disparities in health outcomes that stem from variation in early life microbial exposures.

## MATERIALS AND METHODS

### Participants.

This study was conducted between February and September 2019 in four different populations across two countries, each with a distinct socioeconomic and geographic profile (Table 1). Mothers and infants were recruited opportunistically at four locations: (i) an upper-middle class urban population from Evanston, IL, US (“urban USA”), (ii) an upper-middle class urban population in Xalapa, Veracruz, Mexico (“urban MEX”); (iii) a working-class peri-urban population in the town of Coatepec, Veracruz, Mexico (“peri-urban MEX); and (iv) a rural agricultural population from Ocotepec, Veracruz, Mexico (“rural MEX”). Evanston is a northern suburb of Chicago with a population of around 75,000 people. Xalapa is a major city with a population of almost 480,000 people. Coatepec, a peri-urban town of approximately 92,000, lies 15 kilometers from Xalapa at 1,200 m above sea level, and Ocotepec, a rural agricultural community of approximately 500 people, is 77 kilometers from Xalapa at 2,280 m above sea level. Based on conversations with Mexican collaborators and the authors’ research experience in this region, we anticipated higher environmental contact in infants in the rural MEX population (i.e., more time spent outside) compared to peri-urban MEX, urban MEX, or urban USA. Participants were recruited in the United States using established research registries that are shared between the departments of Psychology and Anthropology at Northwestern University. Participants in Mexico were recruited through word of mouth. All research procedures were approved by the Institutional Review Board of Northwestern University (number STU00210184). Across all populations, consent was obtained by mothers for themselves and their children (in English or Spanish), and verbal consent was acquired in instances where the mother was unable to write. For consistency, we refer to all individuals in this study as “infants,” though the upper end of the age range includes the early stages of toddlerhood.

### Sample collection.

Skin microbiome samples were collected from 25 infants in Evanston, IL, US and 22 infants in Veracruz, Mexico. Exclusion criteria included illness at the time of sample collection (fever, rash, or skin lesion) and current antibiotic use. Across all populations, skin samples were collected from each infant’s right hand (palm), right armpit, and right side of the forehead by rubbing a dry, sterile, dual-tipped cloth swab (Fisher BD BBL Media-free Sterile Swab) on each body site for 1 min. All skin samples were collected by two authors of the study. The US skin samples were collected in an otherwise unoccupied office at a Northwestern University research building in Evanston, IL. In Mexico, skin samples were collected outdoors at three sites: at a school in the urban population, in the waiting area at a health clinic in the peri-urban population, and at a community center in the rural population. To minimize contamination from the sampling environment, swabs were immediately applied to the skin upon removal from the sterile plastic containers. Swabs were returned to the plastic containers immediately after the 1 min of sample collection, at which point the plastic container was stored in a cooler with ice. Contamination of “air microbes” from the sampling areas is unlikely; the microbial biomass of air is low (74, 75), such that studies of microbes in the ambient air typically use pumps to physically capture microbes on filters (75, 79). Control samples were taken from each sample collection location by swirling a swab in the air for 1 min, though without the use of an air pump, this method resulted in control samples that did not amplify during PCR (see below). Samples were stored at −20˚C within a few hours of collection. Samples from Mexico were shipped to Northwestern University on dry ice and immediately stored at −80˚C until DNA extraction.

To supplement biological sample collection, each mother completed a questionnaire that addressed lifestyle factors likely to impact the infant skin microbiome, including those related to delivery mode, household composition, and contact with alloparents (caregivers other than the mother). Mothers also provided data on socioeconomic factors, including age and highest level of education. In the United States, all questionnaires were conducted in English, while in Mexico, all questionnaires were conducted in Spanish. Data from these questionnaires were analyzed in models of infant skin bacterial composition and diversity.

### Microbiome analysis.

DNA was extracted from the skin samples using the Qiagen Powersoil kit at Northwestern University in Evanston, IL, US. Extraction modifications for skin samples included warming the C1 solution, collecting a 750-μl volume of supernatant after incubation in C2, and incubating samples at room temperature after the addition of prewarmed C6 solution. Full extraction protocols can be found in the supplemental materials (Text S1). The V3-V4 region of the 16S rRNA gene was amplified using a modified version of the Earth Microbiome Project protocol (80) and the 515 Fa/926R primer set (81, 82). Skin samples from one infant from the peri-urban MEX population, as well as the three ambient air control samples taken in the field, did not amplify and therefore were not sequenced. We barcoded and pooled amplicons in equal concentrations for sequencing on the Illumina MiSeq V2 platform at the DNA Services Facility at the University of Illinois at Chicago.

10.1128/mSystems.00834-20.7 TEXT S1 DNA extraction protocols. Download Text S1, DOCX file, 0.1 MB. Copyright © 2020 Manus et al. 2020 Manus et al. This content is distributed under the terms of the Creative Commons Attribution 4.0 International license.

Paired-end sequences were joined and processed using QIIME2 v2019.7 (83). Twenty-three control samples (a combination of PCR blanks and negative controls from DNA extraction) were included in the initial data set. Quality filtering and the removal of chloroplast and mitochondria sequences resulted in a total of 4,448,091 reads with an average of 34,622 reads per sample. The dada2 plug-in was used to cluster amplicon sequence variants (ASVs), and taxonomy was assigned by comparing ASVs to the GreenGenes reference database. All samples were rarefied to 10,000 reads per sample based on alpha rarefaction curves, which resulted in the removal of six infant samples, including all three samples from one infant from the peri-urban MEX population.

Twenty control samples were removed during rarefaction, suggesting they had low sequence quality. Three of the control samples (from DNA extraction) remained in the data set after rarefaction, and were dominated by *Enterobacteriaceae* and *Bifidobacterium* spp. The relative abundances of these ASVs in the control samples were much greater than the relative abundances in the infant samples, suggesting that any contamination of the control samples did not significantly skew the ASV abundances in the infant samples. Further, we identified four infant samples in which the relative abundance of *Enterobacteriaceae* was elevated. DNA from these samples was extracted on different days, meaning that any potential contamination event during DNA extraction cannot explain the relative abundance of this ASV in the four infant samples. While *Bifidobacterium* and *Enterobacteriaceae* are typically considered to be members of the gut microbiome community, it is possible that they are also common inhabitants of the infant skin, especially if behaviors like putting hands and objects into the mouth work to transfer microbes from the infant skin to the infant gut (i.e., certain infant “gut microbes” may actually stem from infant skin). For these reasons, we retained the two ASVs in the final data set used in subsequent analyses.

Finally, we also explored statistical trends in the full data set that included the control samples. Nonmetric multidimensional scaling (NMDS) showed that the control samples clearly plotted away from the infant samples and results of permutational analyses of variance (PERMANOVA) revealed significant differences in the bacterial community composition between control and infant samples. For these reasons, we excluded the control samples from subsequent analyses.

Alpha and beta diversity metrics were calculated in QIIME2 on the final data set, which included 119 infant skin samples with a total of 4,332,551 reads and an average of 34,660 reads per sample. Alpha diversity metrics included Faith’s phylogenetic distance and Shannon diversity index, and beta diversity metrics included both unweighted and weighted UniFrac. The full QIIME2 script can be found in Text S2 along with the sample metadata (Data Set S1).

10.1128/mSystems.00834-20.8 TEXT S2 QIIME2 script. Download Text S2, DOCX file, 0.02 MB. Copyright © 2020 Manus et al. 2020 Manus et al. This content is distributed under the terms of the Creative Commons Attribution 4.0 International license.

10.1128/mSystems.00834-20.9 DATA SET S1 Metadata file. Download Data Set S1, XLSX file, 0.1 MB. Copyright © 2020 Manus et al. 2020 Manus et al. This content is distributed under the terms of the Creative Commons Attribution 4.0 International license.

### Statistical analysis.

All statistical analyses were performed in R (84) on the filtered relative abundance table at the bacterial ASV taxonomic level, where an alpha level of 0.05 was used to determine statistical significance. All models controlled for the individual infant. To identify predictors of infant skin bacterial community composition (i.e., beta diversity), we utilized permutational analyses of variance (PERMANOVA) on the unweighted and weighted UniFrac distance matrices using *adonis2* in R. We used both unweighted and weighted UniFrac because the former may capture rare taxa more so than the latter (61). We used pairwise PERMANOVA to identify significant differences in bacterial community composition between the populations, infant skin sites (armpits-foreheads, armpits-hands, and foreheads-hands), and infant ages. Finally, we visualized weighted UniFrac distances by constructing NMDS plots using the *vegan* and *ggplot2* packages.

Independent variables for models of bacterial community composition were chosen in part based on previous studies, as well as our predictions about differences in lifestyle across populations. These variables included: population, skin site, infant’s age (continuous), mother’s age (continuous), mother’s education (categorical: primary, secondary, high school, Bachelor’s, or graduate), household size (continuous), siblings (yes/no), number of alloparents (continuous), and delivery mode (categorical: vaginal or Cesarean section). We used the by=”margin” option in the adonis2 command, which reduces the influence of the order of predictors in PERMANOVA tests. We first calculated the Spearman correlation between predictors and found that mother’s education was correlated with number of alloparents (−0.358) and number of siblings (−0.557). Because any effect of mother’s education was likely mediated by the number of alloparents or siblings, we removed it from subsequent models. We found additional correlations between household size and number alloparents (0.626); population and number of alloparents (0.465); and siblings and household size (0.657), and therefore ran one model that included all seven predictors, and then subsetted the models to tease apart the effects of potentially correlated predictors related to the household (i.e., household size, siblings, and number of alloparents). Results were unchanged when we applied a test to account for false-discovery rate.

We ran ANOVA and Tukey tests on linear mixed effects models, using the package *nlme*, to explore differences in bacterial alpha diversity across populations, skin sites, and infant ages. We subsetted the data into a group of younger infants (0 to 6 months) and a group of older infants (7 to 33 months). These age groups were chosen based on previous observations regarding the development of the skin microbiome (8), including skin physiology (44, 45), as well as to accommodate the uneven distribution of infant ages across the populations in the data set. We ran similar models of ANOVA on linear mixed effects models to test for the influence of particular independent variables on the relative abundance of bacterial taxa at the ASV level. The models were restricted to ASVs with a total abundance count of at least 500 across all samples, and a significance level of 0.01 (after correcting for false discovery) was used. Because 16S rRNA sequencing does not differentiate between bacterial strains, and is limited in its ability to differentiate between bacterial species, we inferred the presence of multiple ASVs of the same name in the relative abundance table as evidence of different bacterial species. For example, if there were multiple “*Rhodobacter*” in the relative abundance table, we manually changed the taxa names to *Rhodobacter1*, *Rhodobacter2*, etc. to reflect the likely scenario that these are different bacterial species and/or strains that are poorly annotated in QIIME2. We also conducted linear discriminant analysis effect size (LEfSe analysis) (85) via the Galaxy web application at the bacterial ASV taxonomic level (LDA score ≥ 3) to identify discriminative bacterial taxa across the populations. Finally, we utilized an algorithm-based random forest classifier to predict the accuracy of classification of samples by body site (test size = 0.6; 72/119 total samples).

### Data availability.

Raw sequence data can be found through SRA using BioProject ID PRJNA669115.
