# Supplementary material for: Infant Skin Bacterial Communities Vary by Skin Site and Infant Age across Populations in Mexico and the United States
Source: mSystems. 2020 Nov 3;5(6):e00834-20. doi: 10.1128/mSystems.00834-20 (PMC7646528; doi:10.1128/mSystems.00834-20)
Supplement: TABLE S1 [file mSystems.00834-20-st001.docx]

*a) Subsetted unweighted UniFrac models:*

*Unweighted UniFrac distance ~ body site + C-section + infant age + siblings + population + (1|individual))*

|  | *df* | *pseudo-F* | *R^2^* | *p-value* |
| --- | --- | --- | --- | --- |
| *Body site* | 2 | 3.607 | 0.053 | **<0.001** |
| *C-section* | 1 | 1.371 | 0.010 | 0.105 |
| *Infant age* | 1 | 3.384 | 0.025 | **<0.001** |
| *Siblings* | 1 | 1.094 | 0.008 | 0.282 |
| *Population* | 3 | 2.930 | 0.064 | **<0.001** |

*Unweighted UniFrac distance ~ body site + C-section + infant age + HH + population + (1|individual))*

|  | *df* | *pseudo-F* | *R^2^* | *p-value* |
| --- | --- | --- | --- | --- |
| *Body site* | 2 | 3.692 | 0.053 | **<0.001** |
| *C-section* | 1 | 1.775 | 0.013 | **<0.05** |
| *Infant age* | 1 | 3.522 | 0.025 | **<0.001** |
| *Household size* | 6 | 1.444 | 0.063 | **<0.01** |
| *Population* | 3 | 2.036 | 0.044 | **<0.001** |

*Unweighted UniFrac distance ~ body site + C-section + infant age + allos + population + (1|individual))*

|  | *df* | *pseudo-F* | *R^2^* | *p-value* |
| --- | --- | --- | --- | --- |
| *Body site* | 2 | 3.532 | 0.050 | **<0.001** |
| *C-section* | 1 | 1.616 | 0.012 | **<0.05** |
| *Infant age* | 1 | 3.897 | 0.028 | **<0.001** |
| *Alloparents* | 1 | 1.525 | 0.063 | **<0.001** |
| *Population* | 3 | 2.058 | 0.044 | **<0.001** |

*b) Subsetted weighted UniFrac models:*

*Weighted UniFrac distance ~ body site + C-section + infant age + siblings + population + (1|individual))*

|  | *df* | *pseudo-F* | *R^2^* | *p-value* |
| --- | --- | --- | --- | --- |
| *Body site* | 2 | 19.120 | 0.227 | **<0.001** |
| *C-section* | 1 | 1.433 | 0.008 | 0.178 |
| *Infant age* | 1 | 3.808 | 0.023 | **<0.01** |
| *Siblings* | 1 | 1.959 | 0.012 | 0.064 |
| *Population* | 3 | 2.986 | 0.053 | **<0.001** |

*Weighted UniFrac distance ~ body site + C-section + infant age + household size + population + (1|individual))*

|  | *df* | *pseudo-F* | *R^2^* | *p-value* |
| --- | --- | --- | --- | --- |
| *Body site* | 2 | 19.924 | 0.228 | **<0.001** |
| *C-section* | 1 | 1.766 | 0.010 | 0.101 |
| *Infant age* | 1 | 3.911 | 0.022 | **<0.01** |
| *Household size* | 6 | 1.836 | 0.063 | **<0.01** |
| *Population* | 3 | 2.680 | 0.046 | **<0.01** |

*Weighted UniFrac distance ~ body site + C-section + infant age + alloparents + population + (1|individual))*

|  | *df* | *pseudo-F* | *R^2^* | *p-value* |
| --- | --- | --- | --- | --- |
| *Body site* | 2 | 19.298 | 0.228 | **<0.001** |
| *C-section* | 1 | 2.035 | 0.012 | 0.064 |
| *Infant age* | 1 | 3.927 | 0.023 | **<0.05** |
| *Alloparents* | 6 | 2.288 | 0.040 | **<0.01** |
| *Population* | 3 | 3.103 | 0.050 | **<0.001** |
