# Supplementary figures and images for: Infant Skin Bacterial Communities Vary by Skin Site and Infant Age across Populations in Mexico and the United States
Source: mSystems. 2020 Nov 3;5(6):e00834-20. doi: 10.1128/mSystems.00834-20 (PMC7646528; doi:10.1128/mSystems.00834-20)

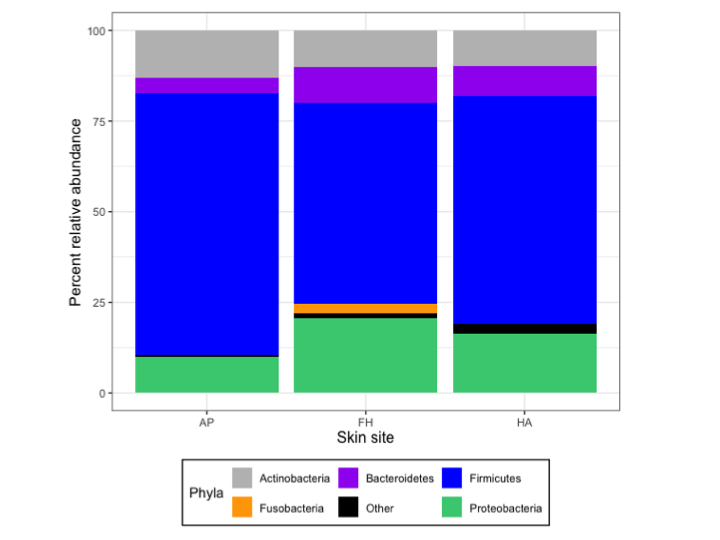

Supplement: FIG S1 [file mSystems.00834-20-sf001.tif]
