# Supplementary material for: Infant Skin Bacterial Communities Vary by Skin Site and Infant Age across Populations in Mexico and the United States
Source: mSystems. 2020 Nov 3;5(6):e00834-20. doi: 10.1128/mSystems.00834-20 (PMC7646528; doi:10.1128/mSystems.00834-20)
Supplement: TABLE S2 [file mSystems.00834-20-st002.docx]

*(a)*

| *All skin sites combined* | *Test estimate* | *p-value* |
| --- | --- | --- |
| urban U.S.A. vs. peri-urban MEX | 0.660 | 0.534 |
| rural MEX vs. peri-urban MEX | 1.904 | **<0.01** |
| urban MEX vs. peri-urban MEX | 0.573 | 0.768 |
| rural MEX vs. urban U.S.A. | 1.244 | **<0.05** |
| urban MEX vs. urban U.S.A. | -0.086 | 0.997 |
| urban MEX vs. rural MEX | -1.330 | 0.060 |

*(b)*

| *Forehead samples* | *Test estimate* | *p-value* |
| --- | --- | --- |
| urban U.S.A. vs. peri-urban MEX | 0.559 | 0.587 |
| rural MEX vs. peri-urban MEX | 3.382 | **<0.001** |
| urban MEX vs. peri-urban MEX | 0.308 | 0.943 |
| rural MEX vs. urban U.S.A. | 2.822 | **<0.001** |
| urban MEX vs. urban U.S.A. | -0.251 | 0.918 |
| urban MEX vs. rural MEX | -3.074 | **<0.001** |

*(c)*

| *Armpit samples* | *Test estimate* | *p-value* |
| --- | --- | --- |
| urban U.S.A. vs. peri-urban MEX | 0.529 | 0.793 |
| rural MEX vs. peri-urban MEX | 0.715 | 0.710 |
| urban MEX vs. peri-urban MEX | -0.837 | 0.793 |
| rural MEX vs. urban U.S.A. | 0.186 | 0.984 |
| urban MEX vs. urban U.S.A. | -1.367 | 0.058 |
| urban MEX vs. rural MEX | -1.552 | 0.075 |

*(d)*

| *Hand samples* | *Test estimate* | *p-value* |
| --- | --- | --- |
| urban U.S.A. vs. peri-urban MEX | 0.518 | 0.919 |
| rural MEX vs. peri-urban MEX | 1.743 | 0.239 |
| urban MEX vs. peri-urban MEX | 2.005 | 0.153 |
| rural MEX vs. urban U.S.A. | 1.224 | 0.269 |
| urban MEX vs. urban U.S.A. | 1.486 | 0.156 |
| urban MEX vs. rural MEX | 0.262 | 0.990 |
