# Supplementary material for: Infant Skin Bacterial Communities Vary by Skin Site and Infant Age across Populations in Mexico and the United States
Source: mSystems. 2020 Nov 3;5(6):e00834-20. doi: 10.1128/mSystems.00834-20 (PMC7646528; doi:10.1128/mSystems.00834-20)
Supplement: TABLE S3 [file mSystems.00834-20-st003.docx]

| *Population* | *All samples combined* | *Hand samples* |
| --- | --- | --- |
| Urban U.S.A. | 1 | 2 |
| Urban MEX | 4 | 21 |
| Peri-urban MEX | 1 | 2 |
| Rural MEX | 30 | 31 |
