# Supplementary material for: Infant Skin Bacterial Communities Vary by Skin Site and Infant Age across Populations in Mexico and the United States
Source: mSystems. 2020 Nov 3;5(6):e00834-20. doi: 10.1128/mSystems.00834-20 (PMC7646528; doi:10.1128/mSystems.00834-20)
Supplement: TABLE S4 [file mSystems.00834-20-st004.docx]

a) Population (N=11 ASVs)

| *ASV* | *rural MEX*  *N=22 samples* | *peri-urban MEX*  *N=14 samples* | *urban MEX N=18 samples* | *urban U.S.A.*  *N=65 samples* | *Test statistic* |
| --- | --- | --- | --- | --- | --- |
| *Thermomonas* | 28.6 | 0.4 | 0.2 | 0.2 | 3.24E-06 |
| *Acinetobacter lwoffii1* | 59.9 | 3.2 | 1.7 | 1.9 | 0.00014 |
| *Janibacter* | 21.7 | 2.3 | 1.8 | 0.2 | 0.00024 |
| *Aerococcus2* | 45.5 | 5.0 | 3.9 | 2.7 | 0.00024 |
| *Arthrobacter1* | 26.9 | 0.4 | 8 | 0.4 | 0.00466 |
| *Staphylococcus9* | 30.1 | 1.8 | 0 | 0 | 0.00466 |
| *Acinetobacter6* | 1.0 | 13.1 | 18.8 | 4.6 | 0.00466 |
| *Brachybacterium2* | 37.2 | 0 | 0 | 0 | 0.00466 |
| *Paracoccus marcusii* | 56.0 | 0.9 | 4.7 | 0.5 | 0.03255 |
| *Streptococcus alactolyticus* | 29.1 | 0 | 0.5 | 0.2 | 0.03792 |
| *Lactococcus2* | 7.4 | 1.6 | 39.8 | 4.5 | 0.03792 |

b) Body site (N=45 ASVs)

| *ASV* | *Hand*  *N=42 samples* | *Armpit*  *N=42 samples* | *Forehead*  *N=35 samples* | *Test statistic* |
| --- | --- | --- | --- | --- |
| *Prevotella14* | 17.8 | 14.7 | 26.3 | 0.00402 |
| *Abiotrophia* | 24.4 | 13.5 | 14.0 | 0.01464 |
| *Actinomyces5* | 14.5 | 9.8 | 14.9 | 0.00402 |
| *Actinomyces6* | 31.9 | 19.1 | 21.1 | 3.87E-05 |
| *Actinomyces7* | 0.09 | 15.4 | 0 | 0.00911 |
| *Anaerococcus8* | 0.1 | 73.8 | 1.4 | 0.03864 |
| *Anaerococcus9* | 24.4 | 210.3 | 5.9 | 0.00182 |
| *Anaerococcus18* | 4.0 | 126.9 | 1.9 | 0.00182 |
| *Corynebacterium12* | 3.3 | 4.9 | 10.1 | 0.00911 |
| *Corynebacterium16* | 119.3 | 350.0 | 19.9 | 1.65E-05 |
| *Corynebacterium kroppenstedtii2* | 4.8 | 7.7 | 17.9 | 0.01464 |
| *Finegoldia6* | 5.2 | 77.4 | 5.9 | 0.00912 |
| *Fusobacterium5* | 48.4 | 51.9 | 101.6 | 0.00912 |
| *Granulicatella1* | 162.1 | 102.5 | 111.5 | 1.65E-05 |
| *Granulicatella4* | 136.6 | 104.6 | 165.0 | 1.89E-06 |
| *Haemophilus4* | 42.1 | 35.6 | 62.3 | 0.00182 |
| *Haemophilus parainfluenzae2* | 48.1 | 29.3 | 34.6 | 0.00911 |
| *Haemophilus parainfluenzae5* | 24.3 | 23.3. | 39.3 | 0.06983 |
| *Haemophilus parainfluenzae6* | 7.3 | 5.1 | 8.0 | 0.02651 |
| *Haemophilus parainfluenzae7* | 6.1 | 5.4 | 10.7 | 0.03863 |
| *Lachnoanaerobaculum orale1* | 6.3 | 5.9 | 10.8 | 0.00912 |
| *Lactococcus2* | 16.2 | 10.0 | 12.7 | 0.03864 |
| *Neisseria1* | 62.3 | 41.3 | 57.9 | 0.00911 |
| *Neisseria4* | 104.9 | 70.5 | 102.2 | 0.01464 |
| *Neisseria subflava3* | 161.6 | 111.7 | 132.2 | 0.01464 |
| *Oribacterium* | 4.6 | 4.6 | 10.4 | 0.03864 |
| *Porphyromonas3* | 41.2 | 41.5 | 80.9 | 6.14E-05 |
| *Porphyromonas4* | 25.6 | 22.0 | 40.7 | 0.00402 |
| *Porphyromonas5* | 72.8 | 51.7 | 76.6 | 0.00912 |
| *Prevotella melaninogenica4* | 80.7 | 56.2 | 81.7 | 0.00912 |
| *Cutibacterium acnes2* | 12.6 | 26.0 | 64.5 | 0.00912 |
| *Cutibacterium acnes3* | 138.6 | 191.7 | 443.6 | 6.14E-05 |
| *Staphylococcus5* | 669.9 | 1759.4 | 346.7 | 4.31E-07 |
| *Streptococcus3* | 371.3 | 199.1 | 205.9 | 0.00182 |
| *Streptococcus8* | 854.1 | 562.7 | 787.5 | 1.65E-05 |
| *Streptococcus13* | 313.3 | 220.5 | 319.8 | 0.00912 |
| *Streptococcus17* | 138.5 | 82.5 | 101.2 | 0.01464 |
| *Streptococcus18* | 116.8 | 77.3 | 97.6 | 0.00075 |
| *Streptococcus22* | 11.6 | 5.5 | 4.8 | 0.00402 |
| *Streptococcus23* | 45.2 | 31.6 | 44.7 | 0.01464 |
| *Streptococcus24* | 42.3 | 24.2 | 25.3 | 0.03864 |
| *Streptococcus infantis1* | 1514.9 | 1045.4 | 1490.4 | 4.11E-06 |
| *Veillonella1* | 107.1 | 73.2 | 104.5 | 0.00402 |
| *Veillonella dispar1* | 143.8 | 88.0 | 96.1 | 0.00044 |
| *Veillonella parvula2* | 37.0 | 24.9 | 32.5 | 0.00402 |

c) Number of alloparents (N=8 ASVs)

| *ASV* | *Test statistic* |
| --- | --- |
| *Peptoniphilus4* | 0.00012 |
| *Acinetobacter3* | 0.00012 |
| *Anaerococcus11* | 0.00012 |
| *Peptoniphilus2* | 0.00012 |
| *Moraxella* | 0.00012 |
| *Staphylococcus5* | 0.00656 |
| *Pseudomonas8* | 0.04286 |
| *Cutibacterium acnes3* | 0.08287 |

d) Household size (N=12 ASVs)

| *ASV* | *Test statistic* |
| --- | --- |
| *Staphylococcus9* | 5.34E-05 |
| *Acinetobacter2* | 5.34E-05 |
| *Staphylococcus21* | 5.34E-05 |
| *Peptoniphilus4* | 0.00023 |
| *Bacillus flexus* | 0.00023 |
| *Anaerococcus11* | 0.00023 |
| *Peptoniphilus2* | 0.00567 |
| *Bacteroides1* | 0.00725 |
| *Tsukamurella* | 0.01846 |
| *Thermomonas* | 0.02996 |
| *Cutibacterium acnes3* | 0.02996 |
| *Streptococcus13* | 0.17523 |
