# Supplementary material for: Infant Skin Bacterial Communities Vary by Skin Site and Infant Age across Populations in Mexico and the United States
Source: mSystems. 2020 Nov 3;5(6):e00834-20. doi: 10.1128/mSystems.00834-20 (PMC7646528; doi:10.1128/mSystems.00834-20)
Supplement: TABLE S5 [file mSystems.00834-20-st005.docx]

| Population | *FH-AP* | *HA-AP* | *HA-FH* |
| --- | --- | --- | --- |
| *Urban U.S.A.* | 5.520, **p<0.001** | 1.387, **p<0.001** | -0.132, p=0.814 |
| *Urban MEX* | 2.637, **p<0.01** | 5.097, **p<0.001** | 2.460, **p<0.01** |
| *Peri-urban MEX* | 0.072, p=0.998 | 1.355, p=0.446 | 1.283, p=0.616 |
| *Rural MEX* | 3.806, **p<0.01** | 2.237, p=0.073 | -1.569, p=0.308 |
