# Supplementary material for: Infant Skin Bacterial Communities Vary by Skin Site and Infant Age across Populations in Mexico and the United States
Source: mSystems. 2020 Nov 3;5(6):e00834-20. doi: 10.1128/mSystems.00834-20 (PMC7646528; doi:10.1128/mSystems.00834-20)
Supplement: TEXT S1 [file mSystems.00834-20-s0001.docx]

**Skin extractions using PowerSoil DNA Isolation Kit (individual columns)**

*Required Equipment and Reagents*

Biosafety cabinet

Microcentrifuge (set to 10,000 rcf *not rpm*)

Pipettes and pipette tips (sterile filter tips)

PowerBead Tubes (one per sample)

Spin Filters (one per sample)

Microcentrifuge Tubes (four per sample plus two additional for C1 and C6)

Heat block or water bath set at 65C

Vortexer

Vortex adapter for 1.5 μl tubes

C1-C6 Solutions

Ethanol

Surgical forceps and scissors

*Prior to starting, label all tubes and then UV all racks, tubes, pipettes, tips, scissors, forceps, etc. in the biosafety cabinet for 15 minutes. Preheat Solution C1 at 65C.*

1. Insert the swab head into the PowerBead Tube. Use sterile forceps and scissors (ethanol and flame sterilization) to cut the swab stem so that the swab head fits into the tube. Repeat for all swabs, re-sterilizing the forceps and scissors in between every sample.
2. Gently vortex to mix.
3. Add 60 μl of Solution C1 to tubes and vortex briefly.
4. Incubate at 65C for 15 mins in heat block or water bath.
5. Secure tubes horizontally on vortexer and vortex at maximum speed for 10 minutes
6. Centrifuge tubes at 10,000 g for 30 sec.
7. Transfer supernatant (up to 600 μl) to clean 2 ml Collection Tube.
8. Add 250 μl of Solution C2 to tubes and vortex for 5 seconds. Incubate at 4C for 5 minutes.
9. Centrifuge the tubes at 10,000 g for 1 min.
10. Transfer up to 750 μl of supernatant to a clean 2 ml Collection Tube
11. Add 200 μl of Solution C3 to the tubes and vortex briefly. Incubate at 4C for 5 minutes.
12. Centrifuge the tubes at 10,000 g for 1 min.
13. Transfer up to 750 μl of supernatant to a clean 2 ml Collection Tube. Be careful to avoid the pellet.
14. Add 1.2 ml of Solution C4 to the supernatant and vortex for 5 sec. Shake Solution C4 to mix prior to adding.
15. Pipette 675 μl of the supernatant to a Spin Filter and centrifuge at 10,000 g for 1 min. Discard the flow through. Repeat until all supernatant has passed through the column
    1. During the first centrifuge cycle, heat required amount of C6 at 65C.
16. Add 500 μl of Solution C5 to the filter and centrifuge at 10,000 g for 30 sec. Discard the flow through.
17. Centrifuge the Spin Filter column a second time at 10,000 g for 1 min.
18. Place Spin Filter into a clean and labeled 2 ml Collection Tube.
19. Add 100 μl of the heated Solution C6 to the center of the filter membrane.
20. Incubate at room temperature for 5 minutes.
21. Centrifuge at 10,000 g for 30 sec.

Discard the spin filter, put the eluted DNA in the freezer.
