# Supplementary material for: Infant Skin Bacterial Communities Vary by Skin Site and Infant Age across Populations in Mexico and the United States
Source: mSystems. 2020 Nov 3;5(6):e00834-20. doi: 10.1128/mSystems.00834-20 (PMC7646528; doi:10.1128/mSystems.00834-20)
Supplement: TEXT S2 [file mSystems.00834-20-s0002.docx]

1. Import data with manifest file

*qiime tools import --type 'SampleData[PairedEndSequencesWithQuality]' --input-path /directory/manifest.csv --output-path /directory/paired-end-demux.qza --input-format PairedEndFastqManifestPhred33*

2. Get summary info

*qiime demux summarize --i-data /directory/paired-end-demux.qza --o-visualization /directory/paired-end-demux.qzv*

3. Quality filter with dada2

*qiime dada2 denoise-paired --i-demultiplexed-seqs /directory/paired-end-demux.qza --p-trunc-len-f 270 --p-trunc-len-r 270 --p-trim-left-f 20 --p-trim-left-r 20 --p-max-ee 6 --p-n-threads 8 --o-table /directory/table.qza --o-representative-sequences /directory/rep-seqs.qza --o-denoising-stats /projects/directory/dada2denoising-stats.qza*

3. Create phylogenetic tree

*qiime phylogeny align-to-tree-mafft-fasttree --i-sequences /directory/rep-seqs.qza --o-alignment /directory/aligned_rep_seqs_dada2.qza --o-masked-alignment /directory/masked_aligned_rep_seqs_dada2.qza --o-tree /directory/unrooted_tree.qza --o-rooted-tree /directory/rooted_tree.qza*

4. Assign taxonomy

*qiime feature-classifier classify-sklearn --i-classifier /directory/gg-13-8-99-nb-classifier-qiime2019-4.qza --i-reads /directory/rep-seqs.qza --o-classification /directory/taxonomy.qza*

5. Filter samples

*qiime taxa filter-table --i-table /directory/table.qza --i-taxonomy /directory/taxonomy.qza --p-exclude mitochondria,chloroplast --o-filtered-table /directory/table.qza*

6. Alpha rarefaction curves

*qiime diversity alpha-rarefaction --i-table /table.qza --i-phylogeny /directory/rooted-tree.qza --o-visualization /directory/alpha_rarefaction --p-max-depth 18000*

7. Diversity metrics

*qiime diversity core-metrics-phylogenetic --i-phylogeny /directory/rooted-tree.qza --i-table /directory/table.qza --p-sampling-depth 10000 --m-metadata-file /directory/metadata.txt --output-dir /directory/core-metrics-results*

8. Extract distance matrices

Weighted UniFrac

*qiime tools extract --input-path /directory/core-metrics-results/weighted_unifrac_distance_matrix.qza --output-path /directory/core-metrics-results/*

Unweighted UniFrac

*qiime tools extract --input-path /directory/core-metrics-results/unweighted_unifrac_distance_matrix.qza --output-path /directory/core-metrics-results/*

9. Export alpha metrics for downstream analyses

Faith’s PD

*qiime tools extract --input-path /directory/core-metrics-results/faith_pd_vector.qza --output-path /directory/core-metrics-results/*

Observed OTUs

*qiime tools extract --input-path /directory/core-metrics-results/observed_otus_vector.qza --output-path /directory/core-metrics-results/*

Shannon

*qiime tools extract --input-path /directory/core-metrics-results/shannon_vector.qza --output-path /directory/core-metrics-results/*

10. Export relative abundance table

*Export taxonomy*

qiime tools export --input-path /directory/taxonomy.qza --output-path /directory/taxonomy.txt

*Export table at ASV level*

qiime tools extract --input-path /directory/core-metrics-results/rarefied_table.qza --output-path /directory/

*Merge taxonomy with feature table*

biom add-metadata -i /directory/feature-table.biom -o /directory/feature-table-taxonomy.biom --observation-metadata-fp /directory/taxonomy.txt --sc-separated taxonomy
